# The electro-structural behaviour of yarn-like carbon nanotube fibres immersed in organic liquids

**DOI:** 10.1088/1468-6996/15/5/055008

**Published:** 2014-10-13

**Authors:** Jeronimo Terrones, Alan H Windle, James A Elliott

**Affiliations:** Department of Materials Science and Metallurgy, University of Cambridge, 27 Charles Babbage Road, Cambridge, CB3 0FS, UK

**Keywords:** carbon nanotube fibres, carbon nanotubes, electrical conductivity, organic liquids, non-ohmic effect, electro-structural phenomena, mathematical model

## Abstract

Yarn-like carbon nanotube (CNT) fibres are a hierarchically-structured material with a variety of promising applications such as high performance composites, sensors and actuators, smart textiles, and energy storage and transmission. However, in order to fully realize these possibilities, a more detailed understanding of their interactions with the environment is required. In this work, we describe a simplified representation of the hierarchical structure of the fibres from which several mathematical models are constructed to explain electro-structural interactions of fibres with organic liquids. A balance between the elastic and surface energies of the CNT bundle network in different media allows the determination of the maximum lengths that open junctions can sustain before collapsing to minimize the surface energy. This characteristic length correlates well with the increase of fibre resistance upon immersion in organic liquids. We also study the effect of charge accumulation in open interbundle junctions and derive expressions to describe experimental data on the non-ohmic electrical behaviour of fibres immersed in polar liquids. Our analyses suggest that the non-ohmic behaviour is caused by progressively shorter junctions collapsing as the voltage is increased. Since our models are not based on any property unique to carbon nanotubes, they should also be useful to describe other hierarchical structures.

## 1. Introduction

Understanding the interactions between carbon nanotube (CNT) fibres [1–4] and their surrounding environment is not only interesting from a scientific standpoint but could also lead to the development of the next generation of energy transmission and storage materials [5–7], high-performance multifunctional composites [8–12], and sensors and actuators [12–16]. Those fibres, directly-spun from a chemical vapour deposition (CVD) reactor [3, 17], or spun from solid arrays of CNTs [4, 18], have highly porous yarn-like structures with accessible specific surface areas ranging from 70 to 200 m^2^ g^−1^ [8, 17, 19]. These relatively high values of porosity mean that properties such as the electrical conductivity and mechanical strength of the fibres are highly dependent on the physical and chemical interactions with their environment.

In previous work [16, 19], we discussed the interactions between direct-spun CNT fibres and several organic liquids. We found that, on immersion, the liquids (i.e. acetone, cyclohexane, cyclohexanone, ethanol, methanol, N-methyl-2-pyrrolidone (NMP), and toluene) infiltrate the fibres, readily filling interbundle pores; however, no evidence of intercalation of liquid molecules inside the bundles was found. The CNT fibres swelled slightly and became less electrically conductive while immersed, but recovered their initial structure and properties when dried. This behaviour was explained qualitatively by reasoning that the energetic cost of generating more CNT-bundle/immersion-medium interface (quantified by the bundle/medium surface energy, 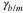) is always lower for the liquids tested than for CNT-bundle/air interface. This means that junctions, formed by bundles bent around obstacles to minimize the total surface energy, may no longer be energetically stable and will spring apart to release the stored elastic energy, resulting in a less interconnected and more electrically resistive CNT network [19]. We also found that if the infiltrating liquids are polar (i.e. acetone, ethanol, methanol, cyclohexanone, cyclohexanol, NMP, and epoxy resin), then the immersed fibres exhibit a non-ohmic effect in which the electrical resistance of the fibre is modulated by an applied electric field. The resistance change is not instantaneous and its rate depends (among other factors) on the viscosity of the immersion medium. We attributed this effect to the accumulation of charge at capacitive interbundle junctions: as the electric field is increased, charge accumulates in open junctions and electrostatic forces bring the bundles closer together, partially reversing the effect of liquid infiltration and thus improving the electrical conductivity of the fibres. The absence of the non-ohmic effect in air and nonpolar liquids (i.e. carbon tetrachloride, cyclohexane, and toluene) was attributed to their smaller dielectric constants, 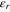, resulting in a tenfold reduction of the strength of electrostatic forces [16].

In this paper, we build upon the qualitative models in our previous work and develop them into simple, physically motivated, mathematical models. We begin by finding expressions for the balance of the elastic and surface energies of a CNT fibre immersed in a medium of particular 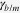, and apply those to estimate the maximum stable length, 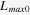, of open bundle junctions in the absence of electric fields. Having found the dimensions of open bundle junctions, we model the effects of electric fields on them, arriving at a way to estimate the stable gap distance, 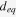, between bundles as a function of applied voltage. We then use our knowledge of 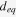 and the hierarchical structure of our fibres to predict the functional relationship between the applied voltage and the electrical resistance of our fibres. Finally, we compare our predictions from different models with experimental data in order to obtain a deeper understanding of the principal structural changes responsible for the non-ohmic effect.

## 2. Structure of direct-spun CNT fibres

**Figure 1.**
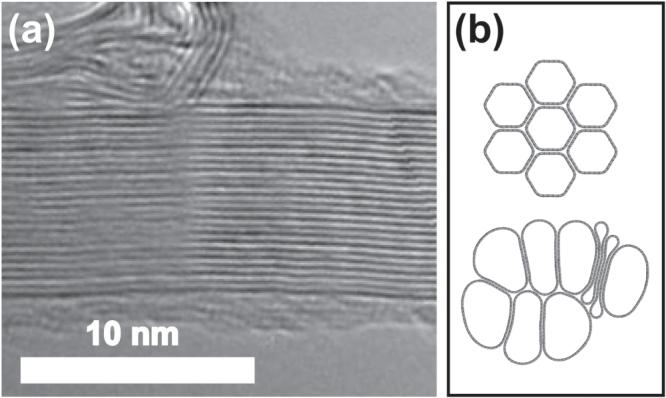
Structure of CNT bundles: (a) transmission electron microscope (TEM) image of a bundle of single-wall carbon nanotubes (SWCNT); (b) schematics of the cross-sections of a bundle of polygonized small diameter SWCNTs (top) and a bundle of larger diameter partially and totally collapsed SWCNTs (bottom).

The physical basis for the models to be constructed in the following discussion is the hierarchical structure of direct-spun CNT fibres. Such fibres are formed in a continuous synthesis process, described in more detail elsewhere [3], by pulling a nanotube aerogel out of the hot zone of a CVD reactor and densifying the extracted material by spray application of a volatile liquid. Depending on the specific synthesis parameters, the fibres may be composed of a variety of nanotubes, from large diameter few-wall tubes to almost exclusively small diameter single-wall tubes [20, 21]. Nevertheless, all samples tested showed a similar behaviour on immersion and removal from organic liquids and a non-ohmic behaviour in polar ones [16]. Carbon nanotubes are known to interact through van der Waals forces and to self-assemble into axially aligned bundles or ‘ropes’ similar to the one shown in figure 1(a). Tubes within a bundle are packed as efficiently as possible to minimize surface energy. For rigid cylinders of identical diameter, the best packing efficiency would be given by a triangular lattice. CNTs, however, have flexible walls and bundles are not usually composed of tubes of the same diameter; this may cause nanotubes to polygonize or collapse, as shown in figure 1(b), in order to minimize the energy of the bundle [22, 23]. According to our previous work, the space between individual nanotubes in a bundle seems to be inaccessible for the liquids we have tested [19].

**Figure 2.**
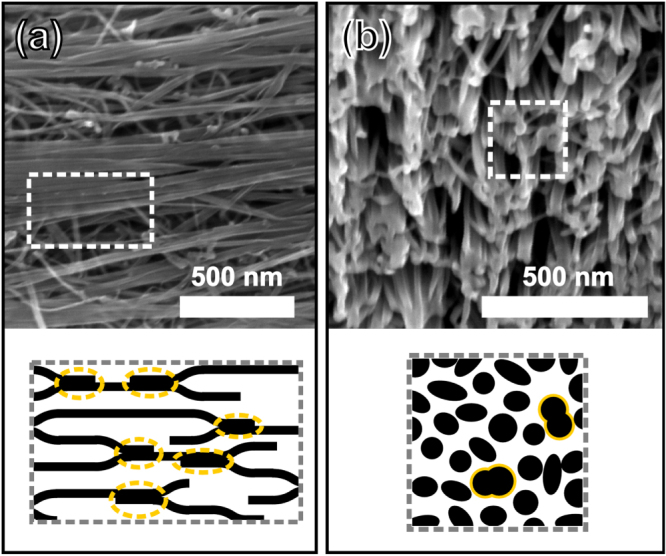
Structure of the CNT bundle network in a direct-spun fibre: (a) scanning electron microscope (SEM) image (top) and schematic (bottom) of the network of CNT bundles that make up a fibre; (b) SEM image (top) and schematic (bottom) of the cross-section of a fibre. Closed interbundle junctions are highlighted in the schematics.

Ideally, CNTs should organize into a single, closely-packed, giant bundle so as to minimize the interfacial surface energy with the surrounding medium; however, this is not the case in practice due to the slow kinetics of nanotube rearrangement during aerogel pull-out and densification, and the presence of impurity particles. The aerogel is thus condensed into a porous hierarchical bundle network, more similar to a staple yarn than a mono-filament fibre [24], such as the one depicted in figure 2. We have evidence that, in contrast to the space between individual CNTs, these contiguous pores are accessed by the immersion media used in previous studies [19], and thus we base our models on structural changes at the *interbundle* level. The number of bundles in a cross-section of the fibre, 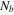, can be estimated by comparing the cross-sectional area of a single bundle to that of the whole fibre (taking porosity into account):

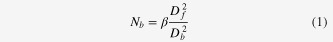
where 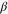 is the packing efficiency (i.e. one minus the porosity) and 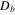 and 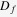 are the diameters of the bundle and the fibre, respectively.

The fibres used in the majority of our previous experiments had diameters around 10 *μ*m, a specific surface area of 75.6 m^2^ g^–1^, a porosity of 54%, and a density of ∼0.6 g cm^−3^. They were composed of nanotube bundles with diameters in the 20 to 30 nm range and elongated pores with diameters in the 30 to 40 nm range [19]. Substituting the relevant numbers into equation (1) gives an estimate of between 51 000 and 115 000 bundles in the cross-section of our fibres, which is used later in section 5 in the calculation of the potential difference across an open junction between CNT bundles in fibre.

## 3. Balance between surface and elastic energies

**Figure 3.**
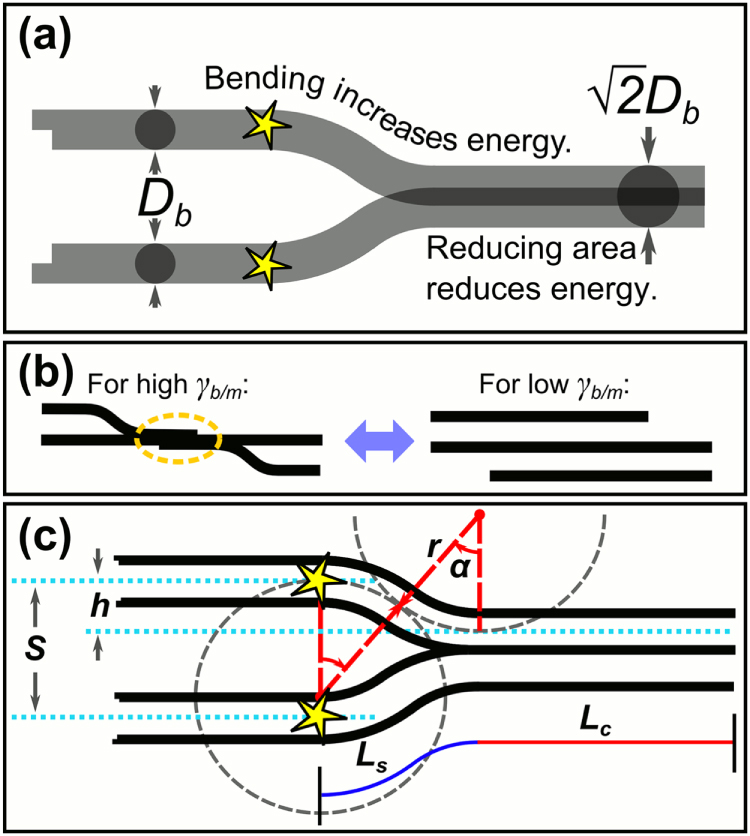
Energy balance model for the CNT bundle network (stars represent entanglement points at which the bundles are fixed): (a) CNT bundles in the fibre will tend to minimize the total energy of the system; bending around obstacles increases elastic energy whereas merging reduces surface energy. (b) Schematic illustrating the possible stable configurations of a set of 3 bundles depending on the specific interfacial energy, 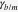, of the surrounding medium. (c) A more detailed schematic of two bundles bending, with definitions of the parameters used in equations (2), (3), (B.2), (B.3), (C.1), and (C.2).

We now develop further our previously proposed qualitative model of junctions opening and closing to minimize the energy of the bundle network [19]. We visualize a closed junction as two cylindrical bundles of diameter 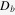 merging into a single bundle of diameter 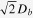 that preserves the volume. This is depicted in figure 3(a), where yellow stars indicate positions where the bundles are fixed due to entanglements or other obstacles. Depending on the value of 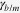, the specific bundle/medium energy, junctions of a particular length will be either closed or open (see figure 3(b)). This means that for each immersion medium there should be a maximum junction length, 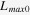, beyond which all bundle junctions are closed. In order to calculate 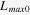, we need to understand the balance between the surface energy, 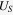, and elastic energy, 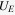. The surface energy can be estimated by:

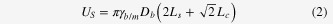
where 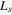 is the length of the portion of the bundles that remain *separated*, bending to bridge the gap distance, and 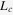 is the length of the *conjoined* region (see figure 3(c)). In an open junction, 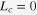 and 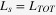, the total length of the junction. Since equation (2) was derived under the assumption that the bundles are smooth, whereas in reality they are composed of several nanotubes and may possibly have a rough surface, it represents a lower bound on the surface area. However, using such an assumption to calculate the specific surface area of our fibres yields a value in good agreement with experimental measurements (see appendix A). Thus, the approximation of smooth bundles seems to hold, at least for systems in which the liquids don’t intercalate inside the bundles. Assuming the bundles to behave as solid beams, which is valid for the small deflections of junctions with a high aspect ratio, the elastic energy stored in a closed junction can be estimated (see appendix B) as:

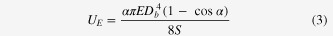
where 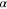 is the angle of curvature (see figure 3(c)), 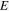 is the elastic modulus, and 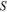 is the separation between the neutral axes of the bundles. For a given total length, 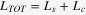, and a set of parameters (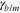, 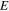, 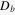, and 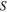), the sum of equations (2) and (3) can be numerically minimized as a function of 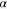 to find the most energetically stable configuration (see appendix C). This can be used to estimate 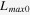 (i.e. the largest 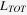 for which an open configuration gives the minimum energy) for the different immersion media used in this study.

For our numerical model, we used the values of 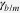 determined in our previous paper [19]; 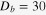 nm; 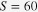 nm, corresponding to two bundles separated by a distance of 30 nm; and 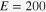 GPa, in accordance with the effective elastic modulus when bending 20–30 nm diameter bundles [25]. Table 1 lists the values of 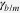 and our calculations of 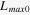 in various immersion media. The values of 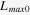 range from 1.5 *μ*m in air to 2.2 *μ*m in NMP. Considering their dependence on 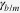, it is not surprising that they correlate well with the previously reported increases in resistance upon immersion [19].

**Table 1.** Values of 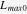 for different immersion media.

Medium	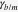 [mJ m^−2^]^a^	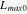 [*μ*m]
Air	47.0	1.49
Ethanol	26.9	1.67
Methanol	25.5	1.69
Acetone	24.6	1.70
Toluene	20.0	1.80
Cyclohexanone	15.4	1.92
NMP^b^	9.6	2.16

a Data from ref. [19].

b N-methyl-2-pyrrolidone.

In our first report of the non-ohmic effect, [16], we compared the strength of the electrodynamic force due to current flowing in the fibre, 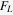, to that of the electrostatic force due to charge being accumulated at interbundle junctions, 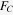. Using a conservative value of 50 nm for the junction length, we found that the electrostatic force was at least three orders of magnitude stronger than the electrodynamic force. Revising this calculation in accordance with our new results we find that, for micrometre-sized junctions, the electrostatic force is in fact at least four orders of magnitude stronger:

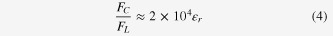
where 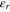 is the relative permittivity (i.e. the dielectric constant) of the medium.

## 4. Behaviour of an open junction under an applied electric field

**Figure 4.**
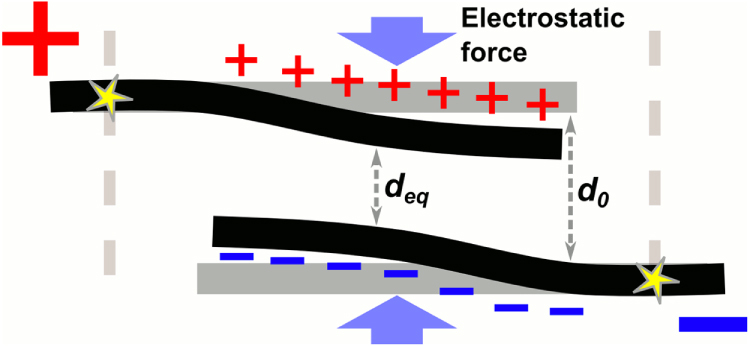
Model of an open interbundle junction under the effect of electrostatic force. As the voltage across the fibre is increased, charge accumulation generates an electrostatic force that bends bundles closer together. The equilibrium distance, 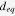, will be that at which the electrostatic force is equal and opposite to the elastic force resulting from bending the bundles.

Figure 4 shows our model of a junction under the effect of an applied field. For each particular voltage, 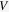, across the junction a certain amount of charge will build up and the bundles will bend until they reach an equilibrium distance, *d_eq_*, at which the electrostatic force is equal and opposite to the elastic force resulting from bending the bundles. To approximate the electrostatic force, we note that the junction resembles a parallel plate capacitor, with 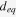 being the separation between the plates. The force for such a system is given by:

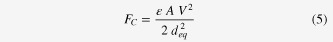
where 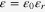 is the permittivity of the medium and 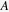 is the area of a plate—a parameter that can be approximated by multiplying the length of the junction times the diameter of a bundle: 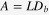. Since the average separation between bundles (∼30 nm) is of the same order as the bundle diameter, in our calculations we account for the curvature of the bundles by adding a small constant to the actual 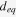 we want to model (see appendix D). For the bending force, one can model the bundles as cantilevers bending under a force applied at the middle from their fixed points (marked by stars in figure 4) and their ends:

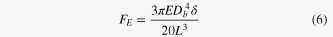
where 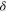 is the deflection of the bundle at its endpoint and 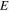 is the elastic modulus. Equating equations (5) and (6) while making 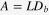 and 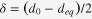, where 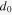 is the (curvature corrected) separation between bundles in the absence of any electric field, yields:

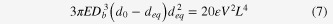
which can be solved for the equilibrium distance.

**Figure 5.**
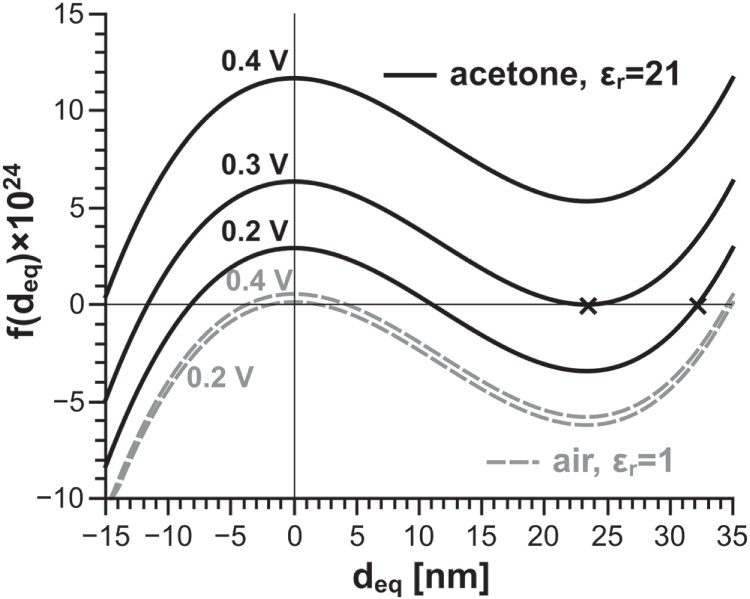
Plots of the function given in equation (8) for a 1 *μ*m long ‘standard’ junction (as defined in text) in different media and at different voltages. The largest positive zeroes of the function correspond to potential energy minima and give the values of 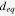 for our model. At high enough voltages, the function has a single or no positive zeroes, indicating that the junction collapses to 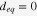.

Equation (7) is cubic in 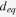 and can be rewritten as:

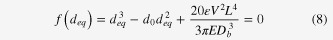
to better show its structure. In figure 5, we plot 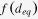 for junctions in acetone and air at several voltages, using the same values for the bundle diameter (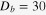 nm), the elastic modulus (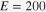 GPa), and junction length (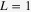 *μ*m), as in our previous calculations, and making 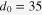 nm to account for the curvature of two round bundles 30 nm apart (see appendix D). This set of parameters (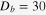 nm, 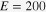 GPa, 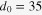 nm) constitutes our ‘standard’ junction and will be used in all our following calculations and plots. The largest positive zeroes of function 8 (marked by crosses in figure 5 for the case of acetone at applied biases of 0.3 V and 0.2 V) give us the values of 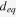 of interest for our models. This can be understood by noting that the forces in equations (5) and (6) come from the negative of the gradient of the potential energy with respect to 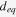:

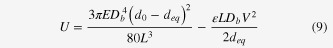



When the function in equation (8) has two positive zeroes, the larger one corresponds to a minimum in the potential energy (i.e. a point of stable equilibrium) whereas the smaller one corresponds to a maximum in the potential energy (i.e. a point of unstable equilibrium). The largest positive zero thus gives the value of 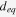 if junction is open. Increasing the voltage across the junction shifts the function *f* (*d_eq_*) upwards along the *y* axis. For sufficiently high voltages, 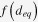 will have no positive zeroes, as in the case for acetone at 0.4 V in figure 5, and the junction is closed. When the function *f* (*d_eq_*) has a single positive zero, the potential energy function (equation (9)) has an inflection point there. This marks the critical voltage beyond which the electrostatic force will be too strong to be opposed by the bending force; which can be physically interpreted as the CNT bundles collapsing into a closed junction.

**Figure 6.**
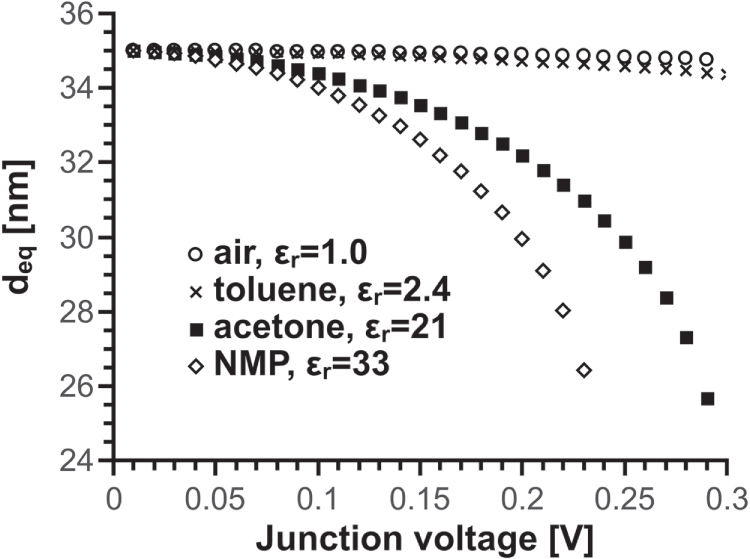
Values of *d_eq_* as a function of voltage for 1 *μ*m long ‘standard’ junctions in different immersion media.

Figure 6 shows the values of 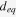 as a function of junction voltage for standard junctions immersed in different media. The dielectric constant of the immersion medium, 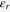, clearly modulates the effects of voltage on the equilibrium distance: for a given voltage, the higher the dielectric constant, the stronger the electrostatic force between bundles and the more they have to bend to oppose it. The change of 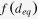 when varying voltage in nonpolar media (air and toluene in the figure) is minimal compared with that in polar liquids (acetone, NMP). This behaviour could account for our observation that the non-ohmic effect does not occur in nonpolar media (i.e. the changes are too small and may be obscured by heating and other effects).

It is possible to use the discriminant of equation (8) to find a function for the maximum junction length that can remain open at a certain voltage (see appendix E):

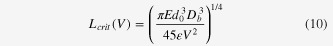



**Figure 7.**
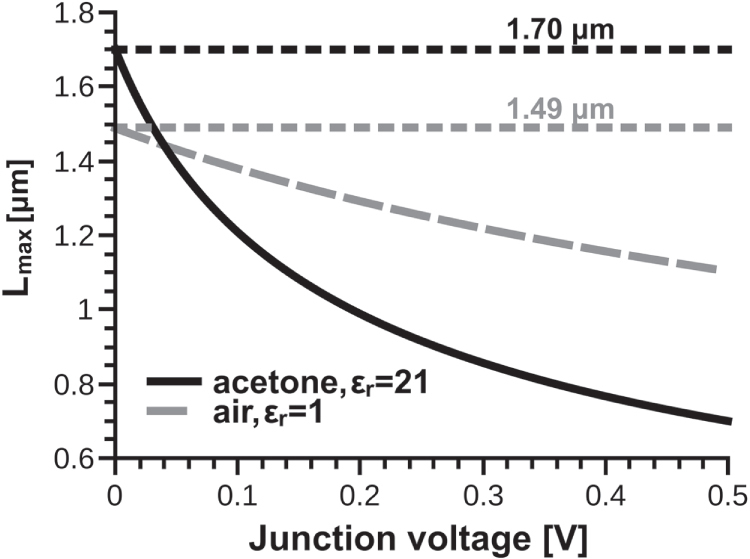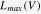 for standard junctions in air and acetone. Horizontal lines indicate the values of 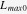.

However, although this gives the correct dependence on voltage for an open junction, it ignores the effect of surface energy and thus indicates that 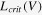 will tend to infinity as the voltage tends to zero. From our calculations in section 3, we know that there is a finite upper bound for the length of an open unpolarized junction immersed in a particular medium, namely 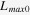. The values of 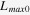 for our standard junction are listed in table 1. To incorporate our knowledge of the effect of surface energy, we perform a change of variable to 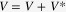 and rewrite:

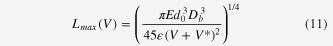
where the constant 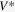 satisfies 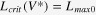. This change shifts the singularity of 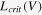 to negative values of 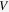 (where it doesn’t affect our model since we are only interested in the voltage difference, which can always be written as a positive number) and allows us to recover 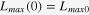. Figure 7 shows the values of 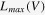 for standard model junctions in air and acetone in the interval 0.0–0.5 V (horizontal lines indicate the values of 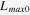). It can be seen that the effects of voltage are less strong in media with a low dielectric constant, in a similar way as was shown in figures 5 and 6, supporting the idea that electro-structural effects are less noticeable in nonpolar media.

It is important to note that the models presented in this section focus on a single isolated junction, ignoring the presence of neighbouring junctions. The electric field of neighbouring junctions is expected to introduce discrepancies in the functional relations we have presented, tending to become less significant as the voltage increases. However, we believe that the analysis of an isolated junction presented here will provide sufficient information on the qualitative behaviour of junctions under applied fields to proceed with the development of our conduction models; any further refinements will be the subject of future publications.

## 5. Models of junction conduction

Our approximate knowledge of the change of 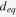 as a function of junction voltage allows us to explore the possible electronic transport mechanisms at the junction and the origin of the non-ohmic effect [16] in our fibres. We will consider two possible conduction mechanisms: diffusive and tunnelling conduction. If the electron transport were diffusive in nature (i.e. following the Drude model), one would expect a linear dependence of resistance on distance:

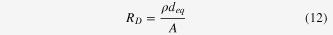
where 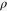 is the resistivity of the liquid. On the other hand, tunnelling conduction would result in an exponential dependence of the form [26]:

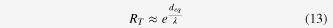
where 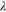 is a characteristic length that depends on the dielectric properties of the junction.

In order to compare these two models to the experimental data, we must first estimate the potential difference across an open junction. We assume that each bundle in the cross-section of a fibre constitutes one of 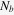 parallel conducting paths running across the fibre. For a sample of finite length, each conducting path has a number, 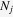, of bundle junctions connected in series along it; of which, for simplicity, in this first model we allow only one junction to be open and assume the rest 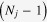 are closed. Under these assumptions, the voltage across the open junction is given by:

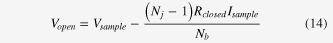
where 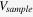 and 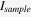 are, respectively, the voltage and current through the entire fibre sample and 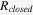 is the resistance of a closed bundle junction. We used equation (14) (taking 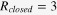 M*Ω*, as reported for thick bundles [27], and 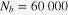) to estimate 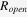 and 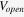, the resistance and voltage of the open junction, from 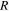 vs 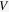 data of real samples immersed in acetone.

**Figure 8.**
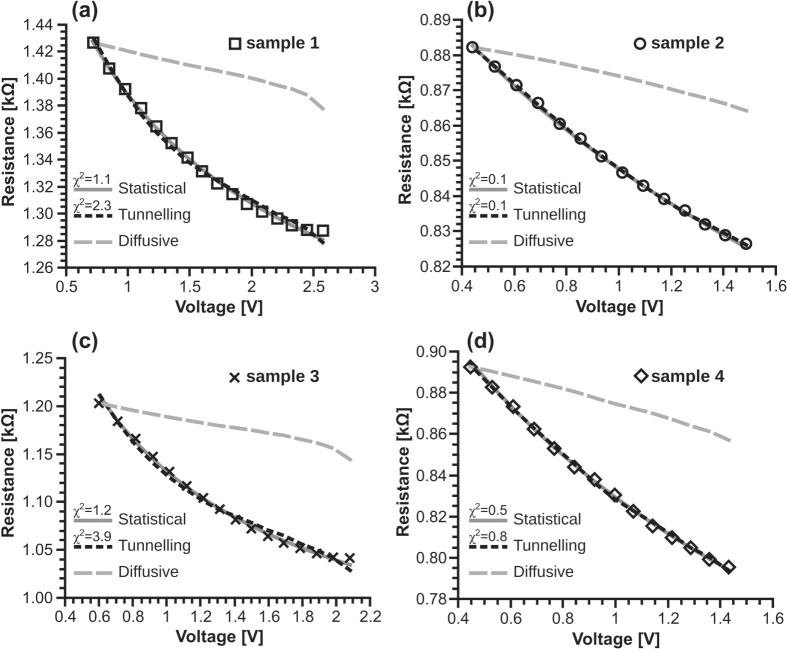
Experimental *R vs V* data of 4 CNT fibre samples immersed in acetone and predictions from 3 different models: tunnelling (dashed black), diffusive (dashed grey), and the ‘statistical’ collapse of bundle junctions (solid grey). The reduced chi-squared calculations for the tunnelling model exclude the last point in (a) and (c) where the model deviates significantly from the data.

We then estimated 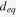 values from a 1 *μ*m standard junction and used a tunnelling-like behaviour, as a function of 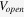, of the form:

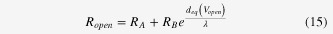
to fit the data. In equation (15), 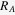 and 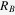 account for the finite value of the resistance when *d*_*eq*_ = 0. Equation (15) was successful in reproducing the resistive behaviour of samples that had previously been cycled several times in the 0.5 mA to 2.0 mA current range to minimize hysteresis; which we consider is related to permanent changes on the fibre as it optimizes its structure [16]. Figure 8 shows experimental data for 4 samples and the predictions obtained by fitting the tunnelling model (black dashed lines), and the alternative models yet to be discussed, to the data. We can see a reasonably good fit, considering all of the simplifications made in the preceding discussion. Moreover, equation (15) qualitatively describes the behaviour of resistance as a function of voltage: which is a monotonically decreasing function with positive curvature. The dashed grey lines in figure 8, on the other hand, show the predictions of the diffusive model, i.e. a linear decrease of 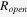 as a function of 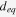, using the same values of 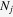 as for the tunnelling fit. Even if the values of 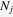 could be tuned to attempt a better fit, it is clear from the shape of the curve, which is monotonically decreasing but with a negative curvature, that the functional form could not correctly describe the resistance change of the fibres even qualitatively. We can therefore reject the diffusive transport model as being the mechanism responsible for conduction at open junctions. So far, it would seem that tunnelling conduction and the reduction of gap distances are responsible for the non-ohmic effect, and we will now examine more closely the consistency and physical reasonableness of the parameters obtained from fitting equation (15) to the data.

Table 2 summarizes the fitting parameters of the relevant models discussed in this work. Values of 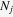 in the range of 13–23 are in good agreement with previous reasoning about the length of bundles and the number of junctions per conductive path in a 1 cm long sample of fibre [16]. From equation (15), we see that the resistance of a collapsed junction, i.e. at 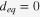, equals 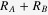. Ideally, this value should be the same as 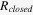, which we took to be 3 M*Ω*. The results from our model are about twice this value but remain on the same order of magnitude. Considering the simplifications in our model, we take this to be a reasonably good agreement, which could perhaps be further improved by tuning the values of 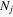 and 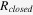. Moving to the next parameter, we notice that the values of 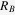 are not consistent; changing by more than 2 orders of magnitude for samples of essentially the same material. Further difficulties arise when we examine the physical soundness of 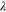, the parameter that describes the sensitivity of the tunnelling resistance to distance. For electrons tunnelling between two electrodes, 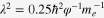, where 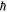 is the reduced Planck’s constant, 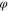 is the work function of the electrodes, and 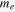 is the effective mass of the electron in the medium between the electrodes. Typical values for 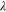 through vacuum or solid dielectrics are of the order of 0.05 nm [26]. The presence of a liquid dielectric between the electrodes significantly reduces the work function of the electrodes and allows for the gap to be crossed by means of multiple tunnelling events through intermediate states [28–30], effectively increasing 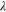 by a factor of ∼10. However, even after considering the enhancement provided by the liquid, the values of 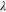 required to fit our experimental data seem rather large. Considering the simplifications we have made in our model, it is possible that we are overestimating the sensitivity of 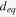 to 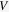 and that the actual changes in distance are smaller than predicted by our model. However it is useful to consider an alternative mechanism that could produce similar macroscopic results without requiring such extreme values of 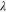 and provide more consistent parameters.

**Table 2.** Fitting parameters for curves from figure 8.

	Tunnelling model			Statistical model	
Sample	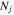	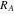 [M*Ω*]	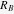 [*Ω*]	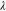 [nm]	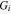 [mS]	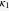 [ 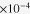 ]	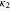
1	23	6.8	17	2.53	0.61	3.4	0.12
2	14	6.4	6	2.54	1.08	5.6	0.05
3	18	6.3	160	2.98	0.64	6.0	0.19
4	13	6.1	2100	4.10	1.02	9.9	0.05

So far, we have been focusing on describing the behaviour of a single standard, 1 *μ*m long, junction and using our models to fit the macroscopic behaviour of the whole sample; a valid approach under the effective medium approximation, developed to explain conduction in disordered materials and random resistor networks [31–33]. Another approach to examine our results is to account for the statistical distribution of different possible lengths of junctions in a fibre. Since we do not have any reason to suppose that there is a preferred junction size, we will assume (in the absence of applied voltage) a uniform distribution of junction sizes, from zero to 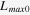 (1.7 *μ*m for acetone). We know from equations (8) to (11) that the effect of an applied potential difference is to close all junctions longer than 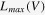. We will now assume that in a highly connected network, well above the percolation limit as CNT fibres are, the increase in conductance (i.e. the reciprocal of resistance) will be proportional to the number of additional closed junctions gained when increasing the voltage. Since we are assuming a uniform distribution of junction lengths, the fraction of closed junctions is given by 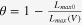, which, with aid of equation (11), can be written as:

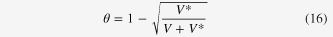
where, as stated in section 4, 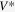 satisfies 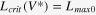. We can now write:

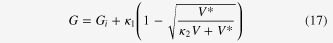
for the conductance, 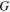, of the fiber. In equation (17), 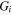 represents the ‘initial’ conductance at zero applied voltage, *κ*_1_ relates the fractional increase of closed junctions to that of conductance, and *κ*_2_ scales the voltage applied to the fibre down to the junction level. The solid grey lines in figure 8 are the predictions from equation (17), where we used 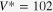 mV, which is the value for our standard junction in acetone, and allowed 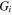, 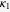, and 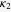 to vary to fit the data. The resulting fitting parameters are listed in table 2.

The statistical model is also able to reproduce the resistive behaviour of the samples. On first glance, figure 8 may appear to show that both of the models, statistical and tunnelling-based, are equally good at fitting the data. However, a closer look at panels (a) and (c) reveals that the tunnelling model deviates to a greater degree, especially at higher voltages. The reduced chi-squared coefficients, 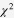, were calculated for both models, assuming an error of ±0.2% in the measurements, and are displayed in figure 8. These coefficients show that both models tend to over-fit the data for relatively small voltage ranges, as shown in panels (b) and (d). For larger voltage ranges, shown in panels (a) and (c) – where the calculation for the tunnelling model already excludes the last data point due to its large deviation, 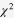 indicates that the model of junctions collapsing as the voltage increases (the ‘statistical’ model) gives a more statistically significant fit than the tunnelling model. The statistical model requires only 3 parameters to be fitted to the data, one fewer than the tunnelling model, which gives it an additional advantage. Furthermore, the parameters used to fit the statistical model (listed in table 2) are more consistent than those for the tunnelling one: with the less stable parameter, 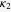, changing by a factor of ∼4 within the samples, a minimal difference when compared with the factor of 350 for 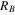 in the tunnelling model. The two proposed mechanisms are not mutually exclusive and it is possible that both of them contribute to the changes in resistance. However, based on the previously listed advantages of the statistical model, and the fact that it doesn’t require unphysically large values of tunnelling length 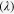 to fit the data, we consider that the progressive collapse of interbundle junctions described by the statistical model is the dominant phenomenon in producing the non-ohmic effect in our direct-spun CNT fibres.

## 6. Conclusions

Using simplified representations of the structure of yarn-like carbon nanotube fibres, we have been able to construct a series of models capable of predicting structural parameters and describe experimentally observed electrical phenomena. The model balancing elastic and surface energies (described in section 3) provides a good explanation for previously reported increases in resistance upon immersion of fibres in organic liquids, [19], and gives predictions of the maximum lengths of open interbundle junctions in such media, which are subject to future experimental confirmation. The ‘statistical’ model, presented at the end of section 5, explains the resistance-voltage functional relationship of the non-ohmic behaviour and points towards its principal structural cause: the progressive collapse of open CNT bundle junctions as voltage is increased. One of the main strengths of these simple models is that they not only reproduce experimental data, as many other curve fits may do, but give an insight on the physics behind the observed phenomena. As the models we constructed are not based on any property unique to carbon nanotubes, they should be useful to predict the electro-structural behaviour of any other hierarchically-structured network of conducting elements.

## Figures and Tables

**Figure D 1.**
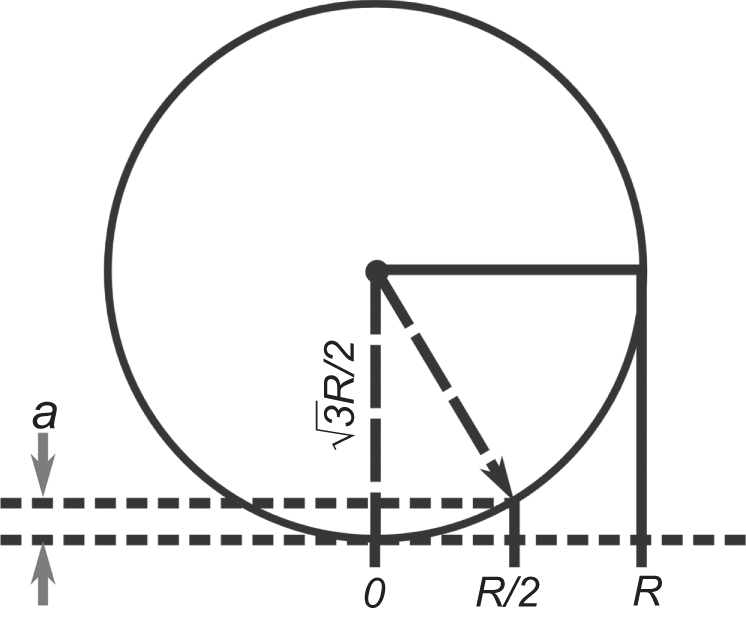
Schematic of the cross-section of an ideally round CNT bundle of radius *R*.

## Appendix A. Estimation of the fibre’s specific surface area

An approximate value for the specific area (in square metres per gram) of the fibre can be obtained from:

where  is the average bundle diameter (expected in nanometres),  is the number of bundles per cross section of fibre,  and is the linear density of the fibre (in units of Tex = grams per kilometre of fibre). Substituting  nm, , and  Tex (a typical value for our fibres) yields  m^2^ g^−1^, which is of the same order of magnitude as experimental results [8, 17, 19]. equation (A.1) assumes the bundles to be solid, smooth cylinders and suggests that the model proposed in section 3 is valid, at least for the case when liquids do not intercalate within bundles.

## Appendix B. Strain energy in a junction

With reference to the schematic in figure 3(c), the strain energy stored in the bent section of a cylindrical beam is given by:

where  is the elastic modulus,  is the second moment of area of the cross-section of the beam,  is the angle of curvature, and  is the length of the bent segment. Substituting  and  in (B.1) we obtain:

where  is the bundle diameter and  is the radius of curvature. Now, for a given ,  and, for the bundles to touch,  (see figure 3(c)). Combining the last two relations and solving for  yields:

Substituting (B.3) in (B.2) and multiplying by 4 (there are 4 bent sections in the junction) yields equation (3).

## Appendix C. Minimizing the energy of a junction

### C.1. Expressing in terms of

The equations:

can be used to put equation (2) terms of  and .

### C.2. Energy minimization routine

Given , , , , and  we used a Matlab routine to find the minimum energy configuration of the interbundle junction. The algorithm first verifies whether ; if not, the bundles in the junction are too short to touch each other even if bent at a straight angle and the minimum energy corresponds immediately to an open junction. If the junction is longer than the minimum length, the algorithm looks for the value of , in the range , that minimizes the sum of equations (2) and (3) and compares such minimum energy to that of an open junction (equation (2) with  and ) to decide whether the minimum energy configuration corresponds to an open or closed junction. The angle  is given by the numerical solution to the transcendental equation , see equation (C.1). For angles smaller than , the value of  becomes negative, meaning that the junction is too short to be closed with such a small angle.

## Appendix D. Accounting for the curvature of bundles in the model for electrostatic force

CNT bundles have a round cross-section and the parallel plate capacitor model assumes a flat surface; using the actual separation between bundles as  would place the electric charges too close together and certainly overestimate the force. To partially mitigate this overestimation, we added a distance  to the separation between bundles. The best way to understand the meaning of  is to refer to the schematic of figure D1 and notice that half of the projected area of the bundle lies below the plane at *a* (measured from the tangent plane) and the other half lies above; the factor of 2 comes from the mirror symmetry of the junction. The value of *a* is then given by:

where  is the radius of the bundle.

## Appendix E. Expression for

The discriminant of the general cubic equation  is given by:

and can be used to determine the nature of the roots of the equation. If , the equation has 3 real roots. If , the equation has only 1 real root (the negative one in the case of equation (8)). Equating (E.1) to zero and solving for  yields equation (10). For a given set of parameters, if  then the discriminant is negative and so is the only root of equation (8), indicating that the bundles collapse to a closed junction.

## References

[C1] Vigolo B, Penicaoud A, Coulon C, Sauder C, Pailler R, Journet C, Bernier P, Poulin P (2000). Macroscopic fibers and ribbons of oriented carbon nanotubes. Science.

[C2] Davis V A (2004). Phase behavior and rheology of SWNTs in superacids. Macromolecules.

[C3] Li Y-L, Kinloch I A, Windle A H (2004). Direct spinning of carbon nanotube fibers from chemical vapor deposition synthesis. Science.

[C4] Zhang M, Atkinson K R, Baughman R H (2004). Multifunctional carbon nanotube yarns by downsizing an ancient technology. Science.

[C5] Zhao Y, Wei J, Vajtai R, Ajayan P M, Barrera E V (2011). Iodine doped carbon nanotube cables exceeding specific electrical conductivity of metals. Sci. Rep..

[C6] Subramaniam C, Yamada T, Kobashi K, Sekiguchi A, Futaba D N, Yumura M, Hata K (2013). One hundred fold increase in current carrying capacity in a carbon nanotube-copper composite. Nat. Commun..

[C7] Cheng H, Dong Z, Hu C, Zhao Y, Hu Y, Qu L, Chen N, Dai L (2013). Textile electrodes woven by carbon nanotube-graphene hybrid fibers for flexible electrochemical capacitors. Nanoscale.

[C8] Vilatela J J, Khare R, Windle A H (2012). The hierarchical structure and properties of multifunctional carbon nanotube fibre composites. Carbon.

[C9] Mora R J, Vilatela J J, Windle A H (2009). Properties of composites of carbon nanotube fibres. Compos. Sci. Technol..

[C10] Lepró X, Ovalle-Robles R, Lima M D, Elías A L, Terrones M, Baughman R H (2012). Catalytic twist-spun yarns of nitrogen-doped carbon nanotubes. Adv. Funct. Mater..

[C11] Dalton A B, Collins S, Muñoz E, Razal J M, Ebron V H, Ferraris J P, Coleman J N, Kim B G, Baughman R H (2003). Super-tough carbon-nanotube fibres. Nature.

[C12] Lima M D (2011). Biscrolling nanotube sheets and functional guests into yarns. Science.

[C13] Slobodian P, Riha P, Lengalova A, Svoboda P, Saha P (2011). Multi-wall carbon nanotube networks as potential resistive gas sensors for organic vapor detection. Carbon.

[C14] Foroughi J (2011). Torsional carbon nanotube artificial muscles. Science.

[C15] Lima M D (2012). Electrically, chemically, and photonically powered torsional and tensile actuation of hybrid carbon nanotube yarn muscles. Science.

[C16] Terrones J, Elliott J A, Vilatela J J, Windle A H (2014). Electric field-modulated non-ohmic behavior of carbon nanotube fibers in polar liquids. ACS Nano.

[C17] Zhong X-H, Li Y-L, Liu Y-K, Qiao X-H, Feng Y, Liang J, Jin J, Zhu L, Hou F, Li J-Y (2010). Continuous multilayered carbon nanotube yarns. Adv. Mater..

[C18] Zheng L (2007). Carbon-nanotube cotton for large-scale fibers. Adv. Mater..

[C19] Qiu J, Terrones J, Vilatela J J, Vickers M E, Elliott J A, Windle A H (2013). Liquid infiltration into carbon nanotube fibers: effect on structure and electrical properties. ACS Nano.

[C20] Gspann T S, Smail F R, Windle A H (2014). FD173: spinning of carbon nanotube fibres using the floating catalyst high temperature route: purity issues and the critical role of sulphur. Faraday Discuss.

[C21] Reguero V, Alemán B, Mas B, Vilatela J J (2014). Controlling carbon nanotube type in macroscopic fibers synthesized by the direct spinning process. Chem. Mater..

[C22] Elliott J, Sandler J, Windle A, Young R, Shaffer M (2004). Collapse of single-wall carbon nanotubes is diameter dependent. Phys. Rev. Lett..

[C23] Motta M, Moisala A, Kinloch I A, Windle A H (2007). High performance fibres from ‘dog bone’ carbon nanotubes. Adv. Mater..

[C24] Vilatela J J, Windle A H (2010). Yarn-like carbon nanotube fibers. Adv. Mater..

[C25] Kis A, Csányi G, Salvetat J-P, Lee T-N, Couteau E, Kulik A J, Benoit W, Brugger J, Forró L (2004). Reinforcement of single-walled carbon nanotube bundles by intertube bridging. Nat. Mater..

[C26] Giaever I, Burstein E, Lundqvist S (1969). Tunneling Phenomena in Solids.

[C27] Nirmalraj P N, Lyons P E, De S, Coleman J N, Boland J J (2009). Electrical connectivity in single-walled carbon nanotube networks. Nano Lett..

[C28] Rostkier-Edelstein D, Urbakh M, Nitzan A (1994). Electron tunneling through a dielectric barrier. J. Chem. Phys..

[C29] Schmickler W (1996). Electronic effects in the electric double layer. Chem. Rev..

[C30] Nitzan A (2001). Electron transmission through molecules and molecular interfaces. Annu. Rev. Phys. Chem..

[C31] Bernasconi J (1973). Electrical conductivity in disordered systems. Phys. Rev. B.

[C32] Kirkpatrick S (1973). Percolation and conduction. Rev. Mod. Phys..

[C33] Erdös P, Haley S (1976). Random-network models of the conductance of disordered condensed matter. Phys. Rev. B.

